# Effect of a Leucine-rich Repeat Kinase 2 Variant on Motor and Non-motor Symptoms in Chinese Parkinson’s Disease Patients

**DOI:** 10.14336/AD.2015.1026

**Published:** 2016-05-27

**Authors:** Qian Sun, Tian Wang, Tian-Fang Jiang, Pei Huang, Dun-Hui Li, Ying Wang, Qin Xiao, Jun Liu, Sheng-Di Chen

**Affiliations:** Department of Neurology & Institute of Neurology, Ruijin Hospital affiliated to Shanghai Jiao Tong University School of Medicine, Shanghai 200025, China

**Keywords:** Parkinson’s disease, *LRRK2*, G2385R, Motor symptoms, Non-motor symptoms

## Abstract

The G2385R variant of the leucine-rich repeat kinase 2 (*LRRK2*) is strongly associated with Parkinson’s disease (PD) in Asian populations. However, it is still unclear whether the clinical phenotype of PD patients with the G2385R variant can be distinguished from that of patients with idiopathic PD. In this study, we investigated motor and non-motor symptoms of *LRRK2* G2385R variant carriers in a Chinese population. We genotyped 1031 Chinese PD patients for the G2385R variant of the *LRRK2* gene, and examined the demographic and clinical characteristics of *LRRK2* G2385R variant carrier and non-carrier PD patients. *LRRK2* G2385R variant carriers were more likely to present the postural instability and gait difficulty dominant (PIGD) phenotype. This variant was also significantly associated with motor fluctuations and the levodopa equivalent dose (LED). G2385R variant carriers had higher REM sleep behavior disorder (RBD) screening questionnaire (RBDSQ) score and more RBD symptoms compared with non-carriers. We concluded that the G2385R variant could be a risk factor for the PIGD phenotype, motor fluctuations, LED values and RBD symptoms.

Parkinson’s disease (PD) is a neurodegenerative movement disorder characterized by classical motor features (resting tremor, rigidity, bradykinesia, and postural instability) and a variety of poorly treatable non-motor symptoms. Although the pathogenesis of this disease, which is the second most common movement disorder, has not been completely delineated, it has been thought to have a multifactorial etiology involving age-related, genetic, and environmental factors [1]. With respect to the genetic component, leucine-rich repeat kinase 2 (*LRRK2*) variants have been recognized as the most common cause of both sporadic and familial PD to date [2]. Of these, the polymorphic variant G2385R has been strongly correlated to PD in the Chinese population, with this variant being observed in 11.9% of PD patients [3], but is absent in Caucasian or Jewish patients.

Genetic association studies have suggested that patients with *LRRK2*-related PD may exhibit some unique phenotypic characteristics, including motor and non-motor symptoms, although there are conflicting reports regarding the clinical presentation [4-7]. Alcalay and colleagues [5] reported that the *LRRK2* G2019S mutation was associated with the postural instability and gait dysfunction dominant (PIGD) phenotype rather than the tremor dominant (TD) phenotype in early onset PD based on the Unified Parkinson’s Disease Rating Scale (UPDRS). However, another large study [4] observed that tremor was the most common motor phenotype among patients with the G2019S mutation, and that non-motor symptoms in PD with the G2019S mutation were more benign than those in idiopathic PD based largely on self-report. Further studies [6, 7] reported no significant correlation of the *LRRK2* G2385R variant with motor phenotype or non-motor symptoms in the Chinese population. Therefore, there appear to be population-specific characteristics in PD patients with *LRRK2* polymorphic variants. So far, the data on the motor and non-motor symptoms of Chinese PD patients carrying the *LRRK2* G2385R variant are still limited [6, 7]. A detailed clinical characterization of PD patients carrying the *LRRK2* variants is warranted.

In the present investigation, we explored the interplay between clinical features and the *LRRK2* G2385R variant genotype in a cohort of Chinese PD patients to determine whether the *LRRK2* G2385R variant influences the development of motor and non-motor phenotypes in this population of PD patients.

## METHODS AND MATERIALS

### Subjects

Patients with PD were recruited from the outpatient clinic at the Department of Neurology, Ruijin Hospital, affiliated to Shanghai Jiao Tong University School of Medicine, Shanghai, China. PD diagnosis was carried out using United Kingdom PD Society Brain Bank criteria [8]. A total of 1031 patients were screened to identify individuals bearing the G2385R variant in the *LRRK2* gene. All the participants provided written informed consent, and the study was approved by the Research Ethics Committee, Ruijin Hospital, affiliated to Shanghai Jiao Tong University School of Medicine, Shanghai, China.

### Genetic analysis

Genomic DNA was extracted from peripheral blood using a standard phenol/chloroform extraction procedure. The *LRRK2* G2385R variant was amplified by PCR with primer pair 5′-TCAATTCAGAATGGTTAGGGAAG-3′ (forward) and 5′-GAAAAGATGGTGCTGAGAAGC-3′ (reverse) as previously described [9]. The PCR products were purified and sequenced on an ABI 3730xl automated sequencer (Applied Biosystems, Foster City, CA, USA).

### Subject evaluation

The following demographic and clinical data were collected from all patients: gender, age, onset age, duration, and medical history. Patient evaluation was carried out by movement disorder specialists who were blinded to the genetic status.

### Motor manifestations

Disease severity was evaluated by the UPDRS and the modified Hoehn and Yahr (H-Y) scale while patients were on treatment. Patients were divided into TD or PIGD subtype, or an intermediate subtype, according to previously described methods [10]. Based on the UPDRS examination scores, patients were subdivided into tremor and PIGD divisions. The tremor division included eight items: self-report of tremor from UPDRS part II, chin tremor, right and left arm tremor, right and left leg tremor, and right and left arm action and postural tremor from UPDRS part III. The PIGD division included five items: self-report of falling, freezing, and walking difficulty from UPDRS part II and gait and postural instability from UPDRS part III. The patients were classified based on the ratio between the summed tremor and PIGD scores as follows: if the ratio was ≥1.5, the participant was classified as belonging to the TD subtype; if ≤1, then to the PIGD subtype; and if between 1.0 and 1.5, then to the intermediate subtype. As the occurrence of motor complications was an important problem in the long-term dopaminergic therapy, we evaluated the prevalence of dyskinesias and motor fluctuations (i.e., predictable wearing-off, unpredictable on-off fluctuations, delayed on effect or sudden off-periods) in PD patients using the UPDRS part IV as previously described [11].

### Non-motor manifestations

Non-motor symptoms were assessed using the Non-Motor Symptoms Questionnaire (NMSQuest) [12]. Autonomic dysfunction was evaluated using the Scale for Outcomes in PD for Autonomic Symptoms (SCOPA-AUT). Odor discrimination was performed as previously described [13] using a 16-item odor identification test, which was an extended version of the “Sniffin’ Sticks” test (SS-16; Burghart Messtechnik, Wedel, Germany), and a cut-off score of 9.5 was taken to be indicative of olfactory dysfunction [14]. The Mini-Mental State Examination (MMSE) [15] was used to investigate cognitive function with a cut-off score of 24 indicating cognitive impairment. The 17-item Hamilton Depression Rating Scale (HAMD-17) was used to evaluate the severity of depressive symptoms [16]; a score of 7 or less was indicative of remission, while higher scores indicated increased severity. The REM sleep behavior disorder (RBD) screening questionnaire (RBDSQ) [17] was used to investigate the RBD symptoms and a score of at least six positive answers was proposed as the cut-off value for clinical probable RBD (cpRBD) [18].

### Statistical analysis

All statistical analyses were performed using the SPSS 16.0 software for Windows (SPSS Inc., Chicago, IL, USA). Data were presented as mean ± standard deviation for numerical variables or numbers, and as a proportion of patients for the categorical variables. The Kolmogorov-Smirnov test was used to verify normal distribution. The T test or Mann-Whitney U test was used as appropriate to compare continuous data with normal distribution or otherwise. The chi-square test, Fisher’s exact test, or the Mann-Whitney U test was used for the comparisons of the categorical variables. An analysis using a covariance model or a logistic regression model was used to evaluate and compare the potential covariates such as age and sex between groups. All statistical tests were two-tailed, and the limit of significance was set at a level of 0.05.

## RESULTS

### LRRK2 G2385R variant prevalence and gender distribution

We genotyped a total of 1031 PD patients (603 males and 428 females) and identified 117 subjects that carried the *LRRK2* G2385R variant (11.3% of total; 61 males and 56 females). There was no significant difference in gender distribution between the *LRRK2* G2385R variant carriers and non-carriers (61 males and 56 females vs. 542 males and 372 females; P = 0.139).

**Table 1:** Comparison of the demographic and motor characteristics of *LRRK2* G2385R variant carrier and non- carrier PD patients

	Whole group (n = 301)	LRRK2 G2385R carriers (n = 76)	LRRK2 G2385R non-carriers (n = 225)	*P* value
Age (years)	62.68 ± 8.39	62.16 ± 8.00	62.85 ± 8.53	0.533
Age at onset (years)	56.68 ± 8.89	55.57 ± 8.78	57.05 ± 8.92	0.208
Sex, Male, n (%)	162 (53.82)	37 (48.68)	125 (55.56)	0.299
Education (years)	10.23 ± 4.61	10.42 ± 4.55	10.16 ± 4.63	0.689
Disease duration (years)	5.94 ± 3.74	6.57 ± 4.21	5.73 ± 3.55	0.177
Hoehn & Yahr stage	2.09 ± 0.81	2.15 ± 0.83	2.08 ± 0.81	0.427
UPDRS-I score	1.73 ± 1.69	1.92 ± 1.98	1.67 ± 1.58	0.301
UPDRS-II score	11.28 ± 4.72	11.16 ± 5.71	11.32 ± 4.35	0.992
UPDRS-III score	26.03 ± 12.51	24.55 ± 12.08	26.52 ± 12.64	0.277
Tremor scores	6.02 ± 3.85	5.24 ± 3.53	6.29 ± 3.92	0.033*
PIGD scores	4.28 ± 2.62	4.88 ± 3.02	4.08 ± 2.44	0.014*
TD phenotype, n (%)	169 (56.10)	29 (38.20)	140 (62.20)	<0.001*
PIGD phenotype, n (%)	112 (37.20)	43 (56.60)	69 (30.70)	<0.001*
Intermediate, n (%)	20 (6.60)	4 (5.30)	16 (7.10)	0.498
Motor fluctuations, n (%)	77 (25.58)	27 (35.53)	50 (22.22)	0.016*
Dyskinesias, n (%)	12 (3.99)	4 (5.26)	8 (3.56)	0.511
LED (mg)	489.41 ± 299.70	553.67 ± 329.84	467.71 ± 286.34	0.016*

UPDRS, Unified Parkinson’s Disease Rating Scale; PIGD, postural instability and gait dysfunction dominant; TD, tremor dominant; LED, levodopa equivalent dose

### Comparison of motor and non-motor phenotypes in variant carriers and non-carriers

Within the *LRRK2* G2385R variant carrier group (n = 117), 38 patients were either referred back to their local physicians or were lost to follow-up. Three other patients who were treated with deep brain stimulation were also excluded. The remaining 76 patients were then further evaluated without knowledge of the PD subtype and clinical characteristics. Simultaneously, 225 patients who did not carry the *LRRK2* G2385R variant were also subjected to further analysis to determine the clinical phenotype.

Demographic and clinical characteristics for the two groups were provided in Table 1 and Table 2. Analysis comparing the two groups revealed no significant differences in patient demographics or in disease severity parameters such as age, sex, education, disease duration, age at onset, H-Y stage and UPDRS scores from part I to part II. However, *LRRK2* G2385R variant carriers showed significantly higher scores for the PIGD division of the UPDRS (4.88 ± 3.02 vs. 4.08 ± 2.44; P = 0.014) and lower scores for the tremor division (5.24 ± 3.53 vs. 6.29 ± 3.92; P=0.033). G2385R variant carriers were more likely to present with the PIGD motor phenotype as compared with non-carriers (56.6% vs. 30.7%; P < 0.001). Conversely, the non-carrier group had lower PIGD scores (4.08 ± 2.44 vs. 4.88 ± 3.02; P = 0.014) and higher tremor scores (6.29 ± 3.92 vs. 5.24 ± 3.53; P = 0.033) than the G2385R variant carrier group; non-carriers also showed a greater prevalence of the TD motor phenotype (62.2% vs. 38.2%; P < 0.001). When compared with non-carriers, the frequency of motor fluctuations (35.53% vs. 22.22%; P = 0.016) and the levodopa equivalent dose (LED) values were also significantly higher in the carrier group (553.67 ± 329.84 vs. 467.71 ± 286.34; P = 0.016; Table 1).

Additionally, analysis of the non-motor symptoms between the two groups revealed that the G2385R variant carrier group had significantly higher mean RBDSQ scores (5.00 ± 3.21 vs. 3.87 ±2.91; P = 0.003) and the number of subjects with a RBDSQ score ≥ 6 (44.74% vs. 28.44%; P = 0.006) than in the non-carrier group, after adjusting for age and sex. Although the mean MMSE score was higher in the G2385R variant carrier group (27.78 ± 2.68 vs. 26.96 ± 2.92; P = 0.034), the frequency of cognitive impairment did not reach statistical significance between the two groups (1.32% vs. 6.22%; P = 0.089). There were no significant differences between the *LRRK2* G2385R variant carriers and non-carriers with respect to the scores for NMSQuest, SCOPA-AUT, SS-16 and HAMD-17. Simultaneously, the frequency of hyposmia, depression and constipation in carriers did not significantly differ from that in non-carriers.

**Table 2:** Comparison of non-motor symptoms in *LRRK2* G2385R variant carrier and non-carrier PD patients

	Whole group (n = 301)	*LRRK2* G2385R carriers (n = 76)	*LRRK2* G2385R non-carriers (n = 225)	*P* value
NMSQuest score	6.17 ± 3.58	6.62 ± 3.56	6.01 ± 3.58	0.143
SCOPA-AUT score	11.29 ± 6.77	11.88 ± 7.12	11.09 ± 6.65	0.220
MMSE score	27.17 ± 2.87	27.78 ± 2.68	26.96 ± 2.92	0.034*
HAMD-17 score	5.49 ± 4.62	6.07 ± 5.05	5.30 ± 4.46	0.263
RBDSQ score	4.16 ± 3.02	5.00 ± 3.21	3.87 ± 2.91	0.003*
SS-16 score^†^	6.83 ± 3.15	6.83 ± 2.97	6.84 ± 3.22	0.796
cpRBD, n (%)	98 (32.56)	34 (44.74)	64 (28.44)	0.006*
Depression, n (%)	85 (28.24)	27 (35.53)	58 (25.78)	0.103
Cognitive impairment, n (%)	15 (4.98)	1 (1.32)	14 (6.22)	0.089
Hyposmia, n (%)	222 (76.82)	59 (77.63)	163 (76.53)	0.767
Constipation, n (%)	156 (51.83)	44 (57.89)	112 (49.78)	0.161

NMSQuest, Non-Motor Symptoms Questionnaire; SCOPA-AUT, Scale for Outcomes in PD for Autonomic Symptoms; MMSE, Mini-Mental State Examination; HAMD-17, 17-item Hamilton Depression Rating Scale; RBDSQ, REM sleep behavior disorder (RBD) screening questionnaire; SS-16, 16-item odor identification test that is an extended version of “sniffin’ sticks” test; cpRBD, clinical probable RBD. ^†^After excluding patients with chronic rhinitis, or who had recently a cold or a nasal operation, 289 patients, including 76 *LRRK2* G2385R variant carriers and 213 non-carriers were applicable for the SS-16 test.

## DISCUSSION

The *LRRK2* G2385R variant is the most commonly occurring pathogenic *LRRK2* substitution in Chinese populations. In our study, this variant was observed at a frequency of 11.3% in PD patients, which was consistent with the results of other studies in Asian populations (range, 5.7-11.9%) [3, 7, 9, 19-22].

Despite extensive study, the role of the *LRRK2* G2385R variant in the development of PD remains unclear. This study was carried out to determine whether the *LRRK2* G2385R variant was associated with unique phenotypic characteristics within a PD patient population. Our findings indicated that while non-carriers showed a higher prevalence of the TD subtype, *LRRK2* G2385R variant carriers were more likely to manifest the PIGD motor subtype, which was associated with an increased mortality risk, accelerated cognitive decline, greater severity of autonomic symptoms, and greater functional disability when compared with the TD phenotype [10, 23-25]. In addition, the frequency of motor fluctuations and the LED values were also significantly higher in the G2385R variant carrier group. Our findings also showed that the mean RBDSQ score and the number of subjects with RBD symptoms were significantly higher in the G2385R variant carrier group than in the non-carrier group.

A growing number of clinical studies have focused on the motor symptoms of *LRRK2*-associated PD, and the literature indicates that *LRRK2* variant carriers mainly manifest the TD motor phenotype [4, 26], which is associated with a more benign clinical presentation compared to the PIGD phenotype [10, 23-25]. However, the motor subtypes identified in these previous studies were not based on the UPDRS scores, and the tremor and PIGD scores were not calculated. In line with the data reported by other groups [5, 27, 28], Alcalay and colleagues [5] conducted examinations based on the UPDRS scores, and found an over-representation of the PIGD subtype in PD patients with the *LRRK2* G2019S mutation compared to non-carriers. Similarly, in the current study, we had performed an extensive observation of the *LRRK2* G2385R variant in association with the PIGD motor phenotype based on the UPDRS scores, and had highlighted critical clinical characteristics of *LRRK2*-related PD in this patient population. We placed special emphasis on the clinical subtype of the *LRRK2* G2385R variant related PD patients because, importantly, the identification of specific motor subgroups could be reflective of disease progression and therapeutic response.

To our knowledge, this is the first report of an association between the G2385R variant and the development of the PIGD motor subtype of PD in a Chinese population. Our findings are not in line with those of previous studies [6, 7]; Wang et al. [6] investigated multiple *LRRK2* variants, including the G2385R and R1628P variant, but did not delineate the clinical characteristics associated with the individual variant. Moreover, this report did not classify the motor subtype based on the calculation of the scores in the TD and PIGD subdivisions. In the other study [7], Gao et al. found no significant difference in motor subtypes when comparing the clinical profiles of 36 carriers and 139 non-carriers; however, it was noteworthy that the tremor score was significantly higher in the non-carrier group.

Previously, Hao et al. [29] and Li et al.[30] reported that in *LRRK2* variant carriers as compared with non-carriers, there were no differences found in the non-motor phenotype through the use of NMSQuest. Our findings supported this result. Nevertheless, considering that NMSQuest was not sensitive enough to detect non-motor symptoms such as autonomic symptoms, olfactory function, cognitive function, depressive and RBD symptoms, we also used SCOPA-AUT, SS-16, MMSE, HAMD-17 and RBDSQ to further investigate associated non-motor symptoms. Our findings showed that the mean RBDSQ score and the number of subjects with the RBD symptoms were significantly higher in the G2385R variant carrier group. As RBD had been associated with a non-tremor predominant phenotype [31, 32], and a higher rate of motor complications [33] in PD patients, our findings may further confirm an important relationship between RBD and motor phenotypes in PD patients with *LRRK2* variants. Although the mean MMSE score was higher in the carrier group, we did not find any significant differences in the frequency of cognitive impairment between carriers and non-carriers. More sensitive examinations are necessary to detect cognitive function and to determine whether the PIGD motor phenotype is associated with cognitive impairment in *LRRK2* variants carriers.

This study has some limitations. Only 76 *LRRK2* G2385R variant carriers and 225 non-carriers were subjected to further analysis, and the analysis of larger samples would be valuable. Additionally, the MMSE may be too insensitive to detect subtle cognitive differences between G2385R variant carriers and non-carriers.

In summary, our study suggested that *LRRK2* G2385R variant carriers present more frequently with the PIGD motor phenotype, motor fluctuations, LED values and RBD symptoms. To further test the association between the *LRRK2* G2385R variant and clinical phenotypes, a longitudinal investigation including a detailed cognitive examination on a larger sample of *LRRK2* G2385R variant carriers is required.

## References

[1] Tolleson CM, Fang JY (2013). Advances in the mechanisms of Parkinson’s disease. Discov Med, 15:61-66. 23375015

[2] Peeraully T, Tan EK (2012). Genetic variants in sporadic Parkinson’s disease: East vs West. Parkinsonism Relat Disord, 18 Suppl 1:S63-65. 22166457 10.1016/S1353-8020(11)70021-9

[3] An XK, Peng R, Li T, Burgunder JM, Wu Y, Chen WJ, et al (2008). LRRK2 Gly2385Arg variant is a risk factor of Parkinson’s disease among Han-Chinese from mainland China. Eur J Neurol, 15:301-305. 18201193 10.1111/j.1468-1331.2007.02052.x

[4] Healy DG, Falchi M, O’Sullivan SS, Bonifati V, Durr A, Bressman S, et al (2008). Phenotype, genotype, and worldwide genetic penetrance of LRRK2-associated Parkinson’s disease: a case-control study. Lancet Neurol, 7:583-590. 18539534 10.1016/S1474-4422(08)70117-0 PMC2832754

[5] Alcalay RN, Mejia-Santana H, Tang MX, Rosado L, Verbitsky M, Kisselev S, et al (2009). Motor phenotype of LRRK2 G2019S carriers in early-onset Parkinson disease. Arch Neurol, 66:1517-1522. 20008657 10.1001/archneurol.2009.267 PMC2837584

[6] Wang C, Cai Y, Gu Z, Ma J, Zheng Z, Tang BS, et al (2014). Clinical profiles of Parkinson’s disease associated with common leucine-rich repeat kinase 2 and glucocerebrosidase genetic variants in Chinese individuals. Neurobiol Aging, 35:725 e1-6. 10.1016/j.neurobiolaging.2013.08.012 24095219

[7] Gao C, Pang H, Luo XG, Ren Y, Shang H, He ZY (2013). LRRK2 G2385R variant carriers of female Parkinson’s disease are more susceptible to motor fluctuation. J Neurol, 260:2884-2889. 24046064 10.1007/s00415-013-7086-9

[8] Hughes AJ, Daniel SE, Kilford L, Lees AJ (1992). Accuracy of clinical diagnosis of idiopathic Parkinson’s disease: a clinico-pathological study of 100 cases. J Neurol Neurosurg Psychiatry, 55:181-184. 1564476 10.1136/jnnp.55.3.181 PMC1014720

[9] Li C, Ting Z, Qin X, Ying W, Li B, Guo Qiang L, et al (2007). The prevalence of LRRK2 Gly2385Arg variant in Chinese Han population with Parkinson’s disease. Mov Disord, 22:2439-2443. 17960808 10.1002/mds.21763

[10] Jankovic J, McDermott M, Carter J, Gauthier S, Goetz C, Golbe L, et al (1990). Variable expression of Parkinson’s disease: a base-line analysis of the DATATOP cohort. The Parkinson Study Group. Neurology, 40:1529-1534. 2215943 10.1212/wnl.40.10.1529

[11] Schrag A, Quinn N (2000). Dyskinesias and motor fluctuations in Parkinson’s disease. A community-based study. Brain, 123:2297-2305. 11050029 10.1093/brain/123.11.2297

[12] Chaudhuri KR, Martinez-Martin P, Schapira AH, Stocchi F, Sethi K, Odin P, et al (2006). International multicenter pilot study of the first comprehensive self-completed nonmotor symptoms questionnaire for Parkinson’s disease: the NMSQuest study. Mov Disord, 21:916-923. 16547944 10.1002/mds.20844

[13] Chen W, Tan YY, Hu YY, Zhan WW, Wu L, Lou Y, et al (2012). Combination of olfactory test and substantia nigra transcranial sonopraphy in the differential diagnosis of Parkinson’s disease: a pilot study from China. Transl Neurodegener, 1:25. 23267690 10.1186/2047-9158-1-25 PMC3582583

[14] Chen W, Chen S, Kang WY, Li B, Xu ZM, Xiao Q, et al (2012). Application of odor identification test in Parkinson’s disease in China: a matched case-control study. J Neurol Sci, 316:47-50. 22364958 10.1016/j.jns.2012.01.033

[15] Folstein MF, Folstein SE, McHugh PR (1975). "Mini-mental state". A practical method for grading the cognitive state of patients for the clinician. J Psychiatr Res, 12:189-198. 1202204 10.1016/0022-3956(75)90026-6

[16] Frank E, Prien RF, Jarrett RB, Keller MB, Kupfer DJ, Lavori PW, et al (1991). Conceptualization and rationale for consensus definitions of terms in major depressive disorder. Remission, recovery, relapse, and recurrence. Arch Gen Psychiatry, 48:851-855. 1929776 10.1001/archpsyc.1991.01810330075011

[17] Stiasny-Kolster K, Mayer G, Schafer S, Moller JC, Heinzel-Gutenbrunner M, Oertel WH (2007). The REM sleep behavior disorder screening questionnaire--a new diagnostic instrument. Mov Disord, 22:2386-2393. 17894337 10.1002/mds.21740

[18] Nomura T, Inoue Y, Kagimura T, Uemura Y, Nakashima K (2011). Utility of the REM sleep behavior disorder screening questionnaire (RBDSQ) in Parkinson’s disease patients. Sleep Med, 12:711-713. 21700495 10.1016/j.sleep.2011.01.015

[19] Wang C, Cai Y, Zheng Z, Tang BS, Xu Y, Wang T, et al (2012). Penetrance of LRRK2 G2385R and R1628P is modified by common PD-associated genetic variants. Parkinsonism Relat Disord, 18:958-963. 22658533 10.1016/j.parkreldis.2012.05.003

[20] Funayama M, Li Y, Tomiyama H, Yoshino H, Imamichi Y, Yamamoto M, et al (2007). Leucine-rich repeat kinase 2 G2385R variant is a risk factor for Parkinson disease in Asian population. Neuroreport, 18:273-275. 17314670 10.1097/WNR.0b013e32801254b6

[21] Kim JM, Lee JY, Kim HJ, Kim JS, Shin ES, Cho JH, et al (2010). The LRRK2 G2385R variant is a risk factor for sporadic Parkinson’s disease in the Korean population. Parkinsonism Relat Disord, 16:85-88. 19854095 10.1016/j.parkreldis.2009.10.004

[22] Fung HC, Chen CM, Hardy J, Singleton AB, Wu YR (2006). A common genetic factor for Parkinson disease in ethnic Chinese population in Taiwan. BMC Neurol, 6:47. 17187665 10.1186/1471-2377-6-47 PMC1764029

[23] Lo RY, Tanner CM, Albers KB, Leimpeter AD, Fross RD, Bernstein AL, et al (2009). Clinical features in early Parkinson disease and survival. Arch Neurol, 66:1353-1358. 19901166 10.1001/archneurol.2009.221

[24] Alves G, Larsen JP, Emre M, Wentzel-Larsen T, Aarsland D (2006). Changes in motor subtype and risk for incident dementia in Parkinson’s disease. Mov Disord, 21:1123-1130. 16637023 10.1002/mds.20897

[25] Allcock LM, Kenny RA, Burn DJ (2006). Clinical phenotype of subjects with Parkinson’s disease and orthostatic hypotension: autonomic symptom and demographic comparison. Mov Disord, 21:1851-1855. 16958096 10.1002/mds.20996

[26] Marras C, Schule B, Munhoz RP, Rogaeva E, Langston JW, Kasten M, et al (2011). Phenotype in parkinsonian and nonparkinsonian LRRK2 G2019S mutation carriers. Neurology, 77:325-333. 21753163 10.1212/WNL.0b013e318227042d PMC3140802

[27] Mirelman A, Heman T, Yasinovsky K, Thaler A, Gurevich T, Marder K, et al (2013). Fall risk and gait in Parkinson’s disease: the role of the LRRK2 G2019S mutation. Mov Disord, 28:1683-1690. 24123150 10.1002/mds.25587

[28] Gan-Or Z, Bar-Shira A, Mirelman A, Gurevich T, Kedmi M, Giladi N, et al (2010). LRRK2 and GBA mutations differentially affect the initial presentation of Parkinson disease. Neurogenetics, 11:121-125. 19458969 10.1007/s10048-009-0198-9

[29] Hao M, Pan N, Zhang Q, Wang X (2014). Mutant of leucine-rich repeat kinase 2 is not associated with non-motor symptoms in Chinese Parkinson’s disease patients. Int J Clin Exp Med, 7:2253-2257. 25232417 PMC4161577

[30] Li DW, Gu Z, Wang C, Ma J, Tang BS, Chen SD, et al (2015). Non-motor symptoms in Chinese Parkinson’s disease patients with and without LRRK2 G2385R and R1628P variants. J Neural Transm, 122:661-667. 25062988 10.1007/s00702-014-1281-4

[31] Kumru H, Santamaria J, Tolosa E, Iranzo A (2007). Relation between subtype of Parkinson’s disease and REM sleep behavior disorder. Sleep Med, 8:779-783. 17904419 10.1016/j.sleep.2007.02.005

[32] Postuma RB, Gagnon JF, Vendette M, Charland K, Montplaisir J (2008). REM sleep behaviour disorder in Parkinson’s disease is associated with specific motor features. J Neurol Neurosurg Psychiatry, 79:1117-1121. 18682443 10.1136/jnnp.2008.149195

[33] Ozekmekci S, Apaydin H, Kilic E (2005). Clinical features of 35 patients with Parkinson’s disease displaying REM behavior disorder. Clin Neurol Neurosurg, 107:306-309. 15885389 10.1016/j.clineuro.2004.09.021

